# Supplementary material for: Investigating microbial population structure and function in the chicken caeca and large intestine over time using metagenomics
Source: BMC Res Notes. 2025 Aug 15;18:355. doi: 10.1186/s13104-025-07441-7 (PMC12357378; doi:10.1186/s13104-025-07441-7)
Supplement: Supplementary file 2 — Recovered nutrient cycles including Carbon, Nitrogen, and Sulphur cycles for all the metagenomic assembled genomes given as PDF images, and identifiable through bin numbers. [file 13104_2025_7441_MOESM2_ESM.zip › Supplementary Data2/bin.115.draw_sulfur_cycle_single.pdf]

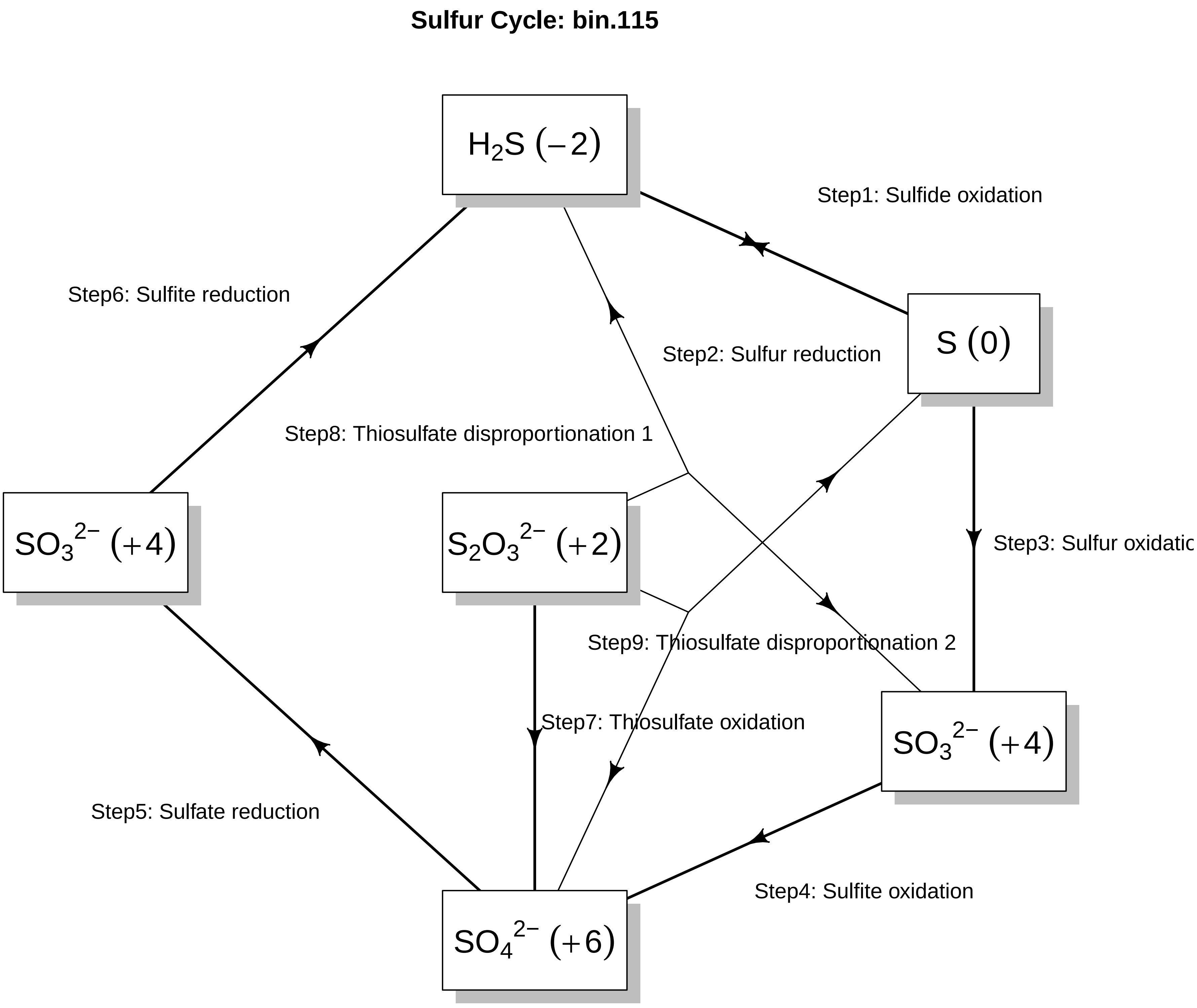

Sulfur Cycle: bin.115
H_2S (−2)
Step1: Sulfide oxidation
Step6: Sulfite reduction
S (0)
Step2: Sulfur reduction
Step8: Thiosulfate disproportionation 1
SO_3^{2-} (+4)
$S_2O_3^{2-}$ (+2)
Step3: Sulfur oxidatic
Step9: Thiosulfate disproportionation 2
Step7: Thiosulfate oxidation
SO_3^{2-} (+4)
Step5: Sulfate reduction
Step4: Sulfite oxidation
SO_4^{2-} (+6)
